# Ya Han Jie ameliorates adjuvant-induced arthritis by inhibiting the NF-κB/NETosis/inflammation axis

**DOI:** 10.1186/s13020-026-01392-2

**Published:** 2026-05-22

**Authors:** Haixu Jiang, Jia Zeng, Enfan Xiao, Jie Xu, Mengdan Wang, Xiaochun Chen, Zixi Huang, Huaxian Pan, Yanru Pan, Yulin Hong, Jie Geng, Qingyi Lu, Guangrui Huang

**Affiliations:** 1 School of Life Sciences, Beijing University of Chinese Medicine, Beijing, China; 2 Department of Rehabilitation, Jiangsu Province People’s Hospital and Nanjing Medical University First Affiliated Hospital, Nanjing, China; 3 School of Rehabilitation Medicine, Nanjing Medical University, Nanjing, China; 4 School of Chinese Pharmacy, Beijing University of Chinese Medicine, Beijing, China; 5 School of Acupuncture & Moxibustion and Tuina, Beijing University of Chinese Medicine, Beijing, China

**Keywords:** Rheumatoid arthritis, *Ya Han Jie*, Neutrophils, NETosis, Dai medicine

## Abstract

**Background:**

Rheumatoid arthritis (RA) is a chronic autoimmune disease that mainly affects the joints. *Ya Han Jie* (YHJ) is a Dai medicine that has significant therapeutic effects on RA. However, the bioactive compounds and potential mechanisms of YHJ in treating RA still need to be elucidated.

**Purpose:**

The aim of this study was to investigate the effects of YHJ on RA and to illuminate its underlying therapeutic mechanisms.

**Methods/study design:**

We injected Freund's complete adjuvant (FCA) into the joint cavity of mice to establish an adjuvant-induced arthritis (AA) mouse model and evaluate the therapeutic effect and safety of YHJ on RA. First, we measured the joint diameters of the mice and assessed the effect of YHJ on the degree of joint swelling. Second, we assessed the effects of YHJ on pathological changes in the joints of AA mice by performing Hematoxylin-eosin staining and Safranin O-fast green staining. Afterward, we used network pharmacology, molecular docking, and molecular dynamics simulations to identify the active components in YHJ that act on RA and explored the therapeutic mechanism of YHJ in treating RA with RNA sequencing. Moreover, we evaluated the effect of YHJ on neutrophil extracellular traps (NETs) using ankle joint tissue sections and purified neutrophils. Finally, we further evaluated the effects of YHJ on the gut microbiota of AA mice by 16S rDNA sequencing.

**Results:**

YHJ significantly reduced joint swelling, arthritis scores, and synovitis-related pathological changes in AA mice. YHJ also inhibited the expression of TNF-α, IL-6, IL-17A, and IFN-γ. UPLC-Q-TOF MS/MS was used to characterize the chemical composition of YHJ, identifying 104 compounds mainly classified as amino sugars, glycosides, alkaloids, and terpenoids. Network pharmacology was employed to identify potential targets and signaling pathways of YHJ in the treatment of RA, and transcriptomic analysis revealed that YHJ downregulated genes associated with NET formation. In vivo and in vitro experiments further confirmed that YHJ significantly inhibited NET formation.

**Conclusion:**

YHJ has a significant therapeutic effect on AA mice by reducing the phosphorylation and activation of NF-κB, decreasing the expression of proteins related to NET formation, and inhibiting NET formation and the release of inflammatory factors in vitro and in vivo.

**Supplementary Information:**

The online version contains supplementary material available at 10.1186/s13020-026-01392-2.

## Highlights

- YHJ showed significant therapeutic effects on adjuvant-induced arthritis in mice.
- The active ingredient YHJ showed high affinity for the docking site of TNF-α.
- YHJ could inhibit the activation of the TNF-α/NF-κB signaling pathway.
- YHJ could inhibit neutrophil extracellular trap formation.

**Supplementary Information:**

The online version contains supplementary material available at 10.1186/s13020-026-01392-2.

## Introduction

Rheumatoid arthritis (RA) is a chronic autoimmune disease that primarily affects joints. It is characterized by inflammation, pain, swelling, and stiffness, which can lead to permanent damage and deformity in severe cases [1]. Repetitive and symmetrical polyarthritis is a key clinical manifestation that is often observed in the hands, wrists, feet, knees and other joints [2]. Early symptoms include redness, swelling, warmth, pain and joint dysfunction [2]. The global prevalence of RA ranges from 0.5% to 1%, decreasing notably from north to south in the Northern Hemisphere and from urban to rural areas [3]. RA affects approximately 1 in 200 adults worldwide and is two to three times more common in women than in men [4]. Although it can occur at any age, the peak incidence lies between 50 and 59 years of age [5]. RA imposes a heavy burden on individuals, mainly through musculoskeletal damage and reduced physical function, which lower their quality of life [6]. In addition to direct medical expenses, the socioeconomic impact arises from the impaired work capacity and decreased social participation of patients [7]. A recent survey in China reported an average yearly direct cost of 1917.21 ± 2559.06 USD per patient [8]. In the United States, approximately 1.5 million patients are affected by arthritis (including osteoarthritis and rheumatoid arthritis), resulting in an annual cost exceeding 200 billion USD [9, 10]. The pathogenesis of RA varies widely among individuals and involves genetic susceptibility, environmental triggers, autoimmune responses, and neutrophil activation [1, 11]. RA can also affect extra-articular tissues and organs such as the eyes, nerves, skin, kidneys, lungs, liver, heart and bones [12–14].

Neutrophils play critical roles in the onset and progression of RA. They can accumulate in inflamed joints by altering chemokine receptor expression and migrating into joint spaces. In patients with RA, neutrophils often exhibit enhanced formation of neutrophil extracellular traps (NETs) [15–17]. These NETs contain citrullinated histones and may serve as a source of autoantigens for rheumatoid autoantibody production [18, 19]. NETs are composed of extracellular strands of nuclear and granular proteins released by activated neutrophils. Their expulsion occurs largely through a specialized cell death process called NETosis [20]. This process begins when microbial products, including endotoxins, bind to neutrophil receptors such as Toll-like receptors (TLRs) and complement receptors, initiating neutrophil activation [21]. Factors such as the size of pathogens and reactive oxygen species (ROS) further regulate NETosis. The subsequent steps involve NADPH oxidase activation and intracellular protease release, followed by histone citrullination and chromatin decondensation [21]. Citrullination, catalyzed by peptidylarginine deiminase 4 (PAD4), reduces the positive charge on core histones by converting arginine residues to citrulline, weakening histone–DNA interactions [20, 21]. The nuclear membrane then ruptures, releasing decondensed chromatin that combines with cytoplasmic and granular proteins—including neutrophil elastase, myeloperoxidase, cathepsin G, the antimicrobial peptide LL-37, high mobility group protein B1 (HMGB1), and protease 3—in the extracellular space [20, 21]. Finally, the integrity of the cell membrane is lost and NETs are released, causing neutrophil death [22]. NET release contributes to the generation of anti-citrullinated peptide antibodies (ACPAs), which in turn stimulate the production of proinflammatory factors (IL-6, IL-8, chemokines, and adhesion proteins), thereby exacerbating RA [19, 23, 24]. Hence, limiting neutrophil accumulation or restraining excessive NET production can be an effective strategy to alleviate or treat RA.

Dai medicine is one of the four major traditional ethnic medical practices in China. Originating from the Dai people in the subtropical region of southwestern China, it integrates indigenous knowledge with influences from ancient Indian and Southeast Asian medical systems. Dai medicine emphasizes the balance of the “four pagodas” (earth, water, fire, and wind) in maintaining health and employs distinctive diagnostic principles and therapeutic approaches. Over centuries, it has accumulated extensive empirical knowledge in treating inflammatory and musculoskeletal disorders, including rheumatoid arthritis (RA). *Ya Han Jie* (YHJ), a classic Dai remedy documented for centuries in *Dang Ha Ya*, consists of four herbal ingredients: Leeae Macrophyllae Folium (*Leea macrophylla* Roxb. ex Hornem.), Lysionoti Herba (*Lysionotus pauciflorus* Maxim.), Ziziphi Oenopoliae Semen (*Ziziphus oenopolia* (L.) Mill.), and Corydalis Rhizoma (*Corydalis yanhusuo* W.T. Wang). In southwestern China, YHJ has substantial clinical benefits [25–28]. Its capsule formulation, Qufengzhitong capsules, is widely used for RA relief and management. Despite its established clinical effectiveness, the chemical basis for the therapeutic action of YHJ remains unclear and warrants further in-depth research.

In our study, YHJ significantly reduced synovial inflammation, cartilage damage and bone erosion in mice with adjuvant-induced arthritis and noticeably decreased joint swelling. In vivo and in vitro experiments showed that YHJ inhibited NET production by suppressing the NF-κB signaling pathway, which regulates neutrophil activity. Notably, the NF-κB pathway is known to affect NET formation by governing key steps in neutrophil activation and the release of extracellular chromatin. In conclusion, this work not only highlights the efficacy of YHJ in RA treatment but also explores its underlying mechanisms, providing a solid experimental basis for its clinical use.

## Materials and methods

### Chemicals and reagents

Ya Han Jie (YHJ) was purchased from Xishuangbanna Banna Pharmaceutical Co., Ltd. (Yunnan, CHN). Methotrexate (MTX) was purchased from SPH Shanghai Sine Pharmaceutical Co., Ltd. (Shanghai, CHN). Freund’s complete adjuvant (FCA) was obtained from Sigma Chemicals (Missouri, USA). The phorbol 12-myristate 13-acetate (PMA) was obtained from Selleck (Texas, USA). The H&E staining kit and a safranin O-fast green staining kit were purchased from Beijing Solarbio Science & Technology Co., Ltd. (Beijing, CHN). A TUNEL staining kit and a PAS staining kit were purchased from Servicebio (Wuhan, CHN). A toluidine blue staining kit was purchased from Beijing Leagene Biotechnology Co., Ltd. (Beijing, CHN). Anti-NE, anti-CitH3 and goat anti-rabbit IgG H&L (Alexa Fluor® 555) secondary antibodies were obtained from Abcam (Cambridge, UK). Anti-IL-6, anti-TNF-α and anti-TNF-R1 antibodies were obtained from Servicebio (Wuhan, CHN). The anti-p-IκBα antibody was obtained from Proteintech (Wuhan, CHN). The anti-p-P65 antibody was obtained from Affinity (Melbourne, AUS). The Opal Multilabel Staining Kit was purchased from AKOYA (Massachusetts, USA). An HRP-conjugated goat anti-mouse/rabbit IgG polymer kit was purchased from ZS GB-Bio (Beijing, CHN). Percoll™ PLUS was purchased from GE Healthcare (Uppsala, SE). The Cytometric Beads Array (CBA) kit was purchased from BD Biosciences (New Jersey, USA). The DAPI reagent was purchased from Keygen Biotech (Jiangsu, CHN).

### Qualitative identification of the chemical components of YHJ

#### UHPLC/MS conditions

The LC-MS analysis was performed on an Agilent 1290 Infinity II UPLC system connected to an Agilent 6545 Q-TOF mass spectrometer (Agilent Technologies, Santa Clara, CA) with an electrospray ionization (ESI) source. YHJ was separated on a Waters XSelect® HSS T3 column (2.1 × 150 mm, 3.5 μm; Waters, Milford, MA, USA) maintained at 35 °C. The mobile phases were acetonitrile (A) and 0.1% formic acid (B), and the gradient elution program was as follows: 0–30 min, 98–2% B, at a flow rate of 0.3 mL·min^−1^. The parameters of the ESI source were set as follows: nebulizer gas pressure, 50 psi; drying gas temperature, 380 °C; drying gas flow rate, 10 L/min; capillary voltage, 3.5 kV; fragment voltage, 135 V; mass range, m/z 150–1500; and collision energy, 30 V. All MS data were analyzed using Xcalibur software.

#### Establishment of molecular networks

The raw MS/MS data were converted to mzXML format using MSConvert. The transformed data were subsequently uploaded to the GNPS Web server (http://gnps.ucsd.edu/), where the molecular network was constructed using an online workflow. Within the GNPS molecular network, the spectral data were carefully reviewed, and those with a match of more than 0.7 were selected. Composite clusters formed by molecular networks were visualized using Cytoscape 3.10.1.

### In vivo evaluation of YHJ efficacy

#### Animals

C57BL/6N mice (male, 7–8 weeks of age) were purchased from Beijing Vital River Laboratory Animal Technology Co., Ltd. [license number: SCXK (jing)-2021-0006]. They were housed in a standard laboratory animal room for one week to acclimate to 12-h light/dark cycle conditions. All experimental procedures were reviewed and approved by the Institutional Animal Care and Use Committee (IACUC) of Beijing University of Chinese Medicine and were performed in accordance with the Guide for the Care and Use of Laboratory Animals. The ethical approval number was BUCM-2021122501-4180.

#### Animal experiments

Seven to eight-week-old mice were used for subsequent experiments after one week of adaptive rearing. As described previously, the mouse adjuvant-induced arthritis (AA) model was induced by injecting Freund’s complete adjuvant (FCA) into the ankle of the left paw on Day 0. Twenty microliters of FCA was injected into the ankle cavity, and 80 μL of FCA was injected around the joint [29]. The negative control mouse ankles were injected with PBS vehicle. The degree of ankle swelling in the mice was subsequently noted, and the pharmacologic intervention was initiated on Day 3. The ankle diameter was assessed every three days with a micrometer (KOGLO, Berlin, Germany). Control (ankle injection and daily oral administration of saline), AA (ankle injection of FCA and oral administration of saline), YHJ (ankle injection of FCA and oral administration of 546 mg·kg^−1^·d^−1^ YHJ), and MTX (ankle injection of FCA and oral administration of 1.73 mg·kg^−1^·2 d^−1^ MTX) groups were established to evaluate the effectiveness of YHJ in treating RA in vivo. Ya Han Jie (YHJ, provided as Chufengzhitong Capsule powder) was used to prepare an aqueous suspension for oral administration, which was then administered orally to mice. The dose of YHJ administered to mice was converted to the dose used in clinical patients.

To further establish the dose–response relationship of YHJ, an independent follow-up animal experiment was conducted under a separate ethical approval (BUCM-20251026-003). In this study, the AA model was induced using Freund’s complete adjuvant from a different commercial source (Chondrex, Cat# 7008), this resulted in a model of greater initial severity, necessitating an extended observation period of 36 days. Mice were randomly divided into 5 groups (*n* = 6 per group): Control, AA model, AA + YHJ-L (273 mg/kg/d), AA + YHJ-H (546 mg/kg/d), and AA + MTX (1.73 mg/kg/2d). All other procedures, including FCA injection, drug administration, and endpoint assessments, were identical to those described for the initial experiment.

#### Assessment of the arthritis score in AA Mice

Throughout the entire experimental phase, the extent of joint inflammation was assessed using a comprehensive 5-tier scoring system [30]. The overall score of the affected limbs can serve as an indicator of the arthritis index for each mouse. For the specific evaluation criteria, please refer to Supplementary Table S1.

#### Collection of specimens

After the mice were anesthetized, their eyeballs were removed to collect blood samples. After centrifugation at 3000 rpm for 30 min, the serum samples were collected and stored at −80 °C until use. The mice were sacrificed by cervical dislocation. Then, ankle tissue samples were collected by excising the ankles of the mice with scissors and removing the excess muscle and skin tissue. After a 48 h incubation in 4% paraformaldehyde, the tissues were transferred to a 10% EDTA decalcification solution (pH 7.2–7.4), and the solution was changed weekly until decalcification was complete.

#### Cytokine analysis

After the mice were anesthetized with isoflurane by inhalation, blood was collected from the mice to determine cytokine levels. The blood was subsequently centrifuged at 3500 rpm for 15 min to obtain the serum. In accordance with the manufacturer's recommendations, the serum was treated with a BD™ Cytometric Bead Array (CBA) Mouse Th1/Th2/Th17 CBA Kit to measure the levels of IL-2, IL-4, IL-6, IL-10, TNF-α, IFN-γ and IL-17A.

#### Quantification of serum NET levels

Circulating NET formation was quantified by measuring myeloperoxidase (MPO)-DNA complexes in mouse serum using a commercial ELISA kit (Zcibio, shanghai, cat#ZC-56424) according to the manufacturer’s instructions. Absorbance was measured at 450 nm using a microplate reader.

#### Histopathological examination

After decalcification, paraffin sections of the mouse joints were prepared as described previously [31]. Hematoxylin-eosin (H&E) staining, toluidine blue staining and safranin O-fast green staining were performed using standard procedures. The procedure was as follows: the slices were immersed in xylene for 10 min, and the process was repeated three times to complete deparaffinization. After deparaffinization, the slices were sequentially immersed in 100%, 95%, 80%, and 70% ethanol for 5 min. Then, the slices were stained with hematoxylin for 30 s and rinsed with running water for 10 min. After the completion of hematoxylin staining, the slices were stained with an eosin solution for 2 min. Then, the slices were sequentially immersed in 80%, 95%, and 100% ethanol for 5 min to complete dehydration. Afterward, the slices were soaked in xylene for 10 min, and this process was repeated twice. Finally, the sections were sealed with neutral resin, and after the neutral resin solidified, the staining levels were observed under a microscope. Toluidine blue staining and safranin O-fast green staining were performed according to the manufacturer's instructions and standard procedures.

To evaluate the potential adverse effects of YHJ on vital organs, liver and kidney tissues were collected from mice in the Control, AA, YHJ, and MTX groups at the endpoint of the in vivo study. The paraffin sections of the above tissues were analyzed with H&E staining, Periodic Acid-Schiff (PAS) staining, and TdT-mediated dUTP nick end labeling (TUNEL) assays according to standard protocols. Stained sections were examined under an optical microscope to assess general morphology, glycogen deposition, basement membrane integrity, and apoptosis.

#### Histological scoring of mouse arthritic joints

Histopathological scoring was performed as previously described [32]. Briefly, the joints of arthritic mice were assigned scores of 0 to 4 for inflammation based on H&E staining according to the following criteria: 0 = normal; 1 = minimal infiltration of inflammatory cells in the periarticular area; 2 = mild infiltration; 3 = moderate infiltration; and 4 = marked infiltration. The joints of arthritic mice were assigned scores of 0 to 4 for bone resorption based on H&E staining according to the following criteria: 0 = normal; 1 = minimal (small areas of resorption, not readily apparent at low magnification); 2 = mild (more numerous areas of resorption in trabecular or cortical bone that were not readily apparent at low magnification); 3 = moderate (obvious resorption of trabecular and cortical bone, without full thickness defects in the cortex; loss of some trabeculae; lesions apparent at low magnification); and 4 = marked (full-thickness defects in the cortical bone and marked trabecular bone loss). Cartilage depletion was identified by toluidine blue staining and safranin O–fast green staining of the matrix and was scored on a scale of 0 to 4, where 0 = no cartilage destruction (full staining with safranin O or toluidine blue), 1 = localized cartilage erosion, 2 = more extended cartilage erosion, 3 = severe cartilage erosion, and 4 = complete depletion of cartilage. Histological analyses were performed in a blinded manner. Images of whole ankles were acquired using a Leica Aperio Versa digital pathology scanner and analyzed using ImageScope (Leica).

#### Immunohistochemistry (IHC)

For IHC, the deparaffinization and hydration procedures were the same as those used for H&E staining. After rehydration, 0.1% Triton X-100 was added, followed by an incubation at 37 ℃ for 20 min to complete permeabilization. Endogenous peroxidase activity was subsequently blocked with 3% hydrogen peroxide. The sections were heated in a pressure cooker to complete antigen retrieval, as described in our previous report [31]. The slices were incubated with 10% goat serum for 30 min at 37 ℃. Appropriate concentrations of the primary antibodies were applied. After an overnight incubation at 4 ℃, the corresponding horseradish peroxidase (HRP)-labeled secondary antibodies were incubated with the slices at 37 ℃ for 30 min. 3,3’-Diaminobenzidine (DAB) was used to detect positive signals. Hematoxylin was used to stain the cell nucleus. The subsequent procedures were the same as those used for H&E staining. Positive signals were analyzed using ImageJ software.

#### Western blot analysis

Protein was extracted from snap-frozen ankle joint tissues using RIPA lysis buffer containing protease and phosphatase inhibitors. Equal amounts of protein were separated by SDS-PAGE and transferred to PVDF membranes. After blocking, membranes were incubated overnight at 4 °C with primary antibodies against PAD4(Proteintech, cat#17,373-1-AP), STAT3(CellSignaling, cat#9139), and β-actin(Selleck, cat#F0012). Following incubation with HRP-conjugated secondary antibodies, protein bands were visualized using an enhanced chemiluminescence (ECL) detection system. Densitometric analysis was performed using ImageJ software.

### In vitro evaluation of YHJ efficacy

#### Preparation and culture of neutrophils

C57BL/6 mice (7–8 weeks of age) were intraperitoneally injected with 1 mL of 10% peptone, and 12 h later, 1 mL of the solution was injected again. After the mice were sacrificed, 5 mL of RPMI-1640 medium containing 10% fetal bovine serum (FBS) and 1% antibiotics was injected into the peritoneal cavity to obtain lavage fluid. After centrifugation, the cell pellet was resuspended in 1 mL of RPMI-1640 medium and placed in a discontinuous Percoll gradient separation solution (54.8%/70.2%) to purify the neutrophils [33]. Cells in the lavage fluid were stratified on a Percoll gradient and centrifuged at 1500 × g for 30 min at 22 °C. After centrifugation, the lower interface contained purified neutrophils. Afterwards, flow cytometry was used to test the purity of the isolated mouse peritoneal neutrophils. Then, 1 mL of RPMI-1640 (Corning, NY, USA) containing 10% FBS (Corning, NY, USA) was used to culture the neutrophils with 5% CO_2_ at 37 °C.

#### Preparation of YHJ-containing serum

Sprague–Dawley (SD) rats were adaptively fed in the animal laboratory for one week and randomly divided into two groups, with 7 rats in each group: the normal serum group and the YHJ drug-containing serum group. YHJ was prepared from Qufengzhitong capsules and ground evenly in a mortar. The YHJ solution was then prepared with water.

The YHJ-containing serum group was gavaged with YHJ (273 mg/kg), and the normal serum group was gavaged with the same amount of saline for 7 days. One hour after the administration of the last dose, the rats were anesthetized with isoflurane by inhalation, and blood was collected from the abdominal aorta and centrifuged at 3500 rpm for 15 min. Afterward, the serum was processed at 56 °C for 30 min and filtered through a 0.22 μm microporous membrane for sterilization. Finally, the serum samples were stored at −80 °C for subsequent experiments.

#### Gain-of-function validation of the TNF-α/NF-κB axis in NETosis

To directly validate the functional role of the TNF-α/NF-κB signaling axis in promoting NETosis, a gain-of-function experiment was performed. Isolated mouse peritoneal neutrophils were resuspended in RPMI-1640 base medium at a density of 5 × 10^5^ cells/mL. The cells were then seeded onto poly-D-lysine (PDL)-coated coverslips pre-placed in 24-well plates and allowed to adhere for 1 h under standard culture conditions (37°C, 5% CO₂). After the adherence period, the cells were divided into four treatment groups: Control group: No additional stimulation. TNF-α group: Stimulated with 50 ng/mL recombinant mouse TNF-α protein (MedChemExpresss, cat#HY-P7090). PMA group: Stimulated with 100 nM PMA (Sigma-Aldrich, Cat#P1585). PMA + TNF-α group: Co-stimulated with 100 nM PMA and 50 ng/mL recombinant mouse TNF-α protein.

After a 4-h incubation, NET formation was assessed by immunofluorescence staining for citrullinated histone H3 (CitH3) as previously described. The fluorescence intensity of CitH3 was quantified using ImageJ software across multiple random fields to serve as a quantitative indicator of NETosis.

### Network pharmacology combined with a transcriptomic analysis

#### Target prediction

The components in YHJ were input into the SwissADME (GI absorption = High, http://www.swissadme.ch/) online tool to evaluate the gastrointestinal absorption potential of each component [34, 35]. The components that could be absorbed by the gastrointestinal tract were used as potential compounds to predict targets in the SwissTargetPrediction database (with a probability threshold set at > 0.4, http://www.swisstargetprediction.ch/)[36]. For targets related to RA, data were obtained from the DisGeNET database (https://disgenet.com/) [37]. The Venn tool was used to merge the potential compound targets and RA-related targets to identify common targets.

#### Network establishment

The identified YHJ targets were input into the STRING database (https://www.string-db.org/) [38] to generate a protein–protein interaction (PPI) network with a high confidence threshold set at 0.7. We constructed a YHJ–compound–target–RA network. The resulting networks were visually analyzed using Cytoscape 3.10.1.

#### GO functional enrichment analysis and KEGG pathway enrichment analysis

GO functional annotation and KEGG pathway enrichment analyses were performed using the DAVID database (https://david.ncifcrf.gov/), and the top 20 items were screened based on fold enrichment values to identify the signaling pathways and mechanisms of YHJ treatment for RA.

#### Molecular docking

We identified the relationships between the potential targets and active ingredients of YHJ for RA treatment by combining the results of the "YHJ–compound–target–RA" network and the PPI network, and the key active compounds of YHJ and the key potential targets for RA therapy were subjected to molecular docking using AutoDock Vina [39]. The crystal structures of TNF-α (ID: 6X81), STAT3 (ID: 6NJS), PTGS2 (ID: 5IKR), CXCR4 (ID: 8K3Z), and IL2 (ID: 1M48) were obtained from the Protein Data Bank (http://www.rcsb.org/). Ligands were preprocessed by AutoDock Tools [40]. The final conformations in the docking simulation were checked and presented using PyMOL software.

#### Molecular dynamic simulation

Molecular dynamic (MD) simulations are widely used to explain molecular interactions through certain trajectories. Here, MD simulations were performed to further verify the rationality and reliability of the docking results. They were conducted using GROMACS 2020 software for 100 ns trajectories [41]. The ACPYPE online server was utilized to generate topological structures for small molecules [42]. The CGenFF force field was applied, and an AM1-BCC charge was added to create a topology file for subsequent simulations. The topological structure of the protein was constructed using CHARMM36 force field parameters. Each interaction system was solvated in a tetrahedral box using the TIP3P water model, while the protein and box boundaries were maintained at a minimum distance of 1.0 nm. All interaction systems were automatically neutralized using a generation tool with the addition of chloride or sodium ions. In addition, the steepest descent algorithm was used to minimize the energy of these systems. After the ligand coordinate position was limited, NVT (constant particle number, volume, and temperature) and NPT (constant particle number, pressure, and temperature) equilibria were performed at 100 ps under a 1.0 bar atmosphere and 300 K conditions, respectively. Finally, a 100 ns MD simulation of each system was performed, and the coordinate trajectory was recorded every 10 ps [43–45].

### RNA sequencing

At the end of the animal experiments, the mice were anesthetized with isoflurane and sacrificed. Afterward, the femurs and tibias of the mice were rinsed with precooled PBS to obtain bone marrow cells. Finally, bone marrow neutrophils were obtained by flow cytometry sorting for subsequent experiments. Total RNA was isolated from the bone marrow neutrophils of the control, AA and YHJ-treated mice using TRIzol reagent and then reverse transcribed to prepare cDNA libraries. Clean data were mapped to the reference database GRCm38. The expression level of RNAs was calculated as fragments per kilobase of exon per million fragments mapped (FPKMs). Differentially expressed genes between different groups were determined using an “|Fold Change|≥ 2, adjusted *P* value < 0.05” as the threshold for significance. The functional pathway analysis was performed using the KEGG database. To generate new geneclusters from RNA-seq data, we used the R package Mfuzz (v2.60.0) in conjunction with the k-means algorithm [46], determining the optimal number of clusters using the “get clusters” function. For KEGG pathway and GO enrichment analyses, we used the R package ClusterGVis (v0.1.0), setting adjusted *P* < 0.05 as a significant threshold for enrichment [47].

### Statistical analysis

All statistical analyses were performed using GraphPad Prism version 10.0 (GraphPad Software, San Diego, CA, USA). Data are expressed as mean ± standard deviation (SD). Normality and homogeneity of variances were assessed prior to statistical testing. For time-course data, two-way ANOVA followed by Tukey's post hoc test was used to assess the overall effects of treatment and time. For comparisons at a single time point, one-way ANOVA with appropriate post hoc tests was applied. For two-group comparisons, an unpaired two-tailed Student's t-test was used. A p-value of less than 0.05 was considered statistically significant.

## Results

### YHJ improves symptoms of RA in AA mice

**Fig. 1:**
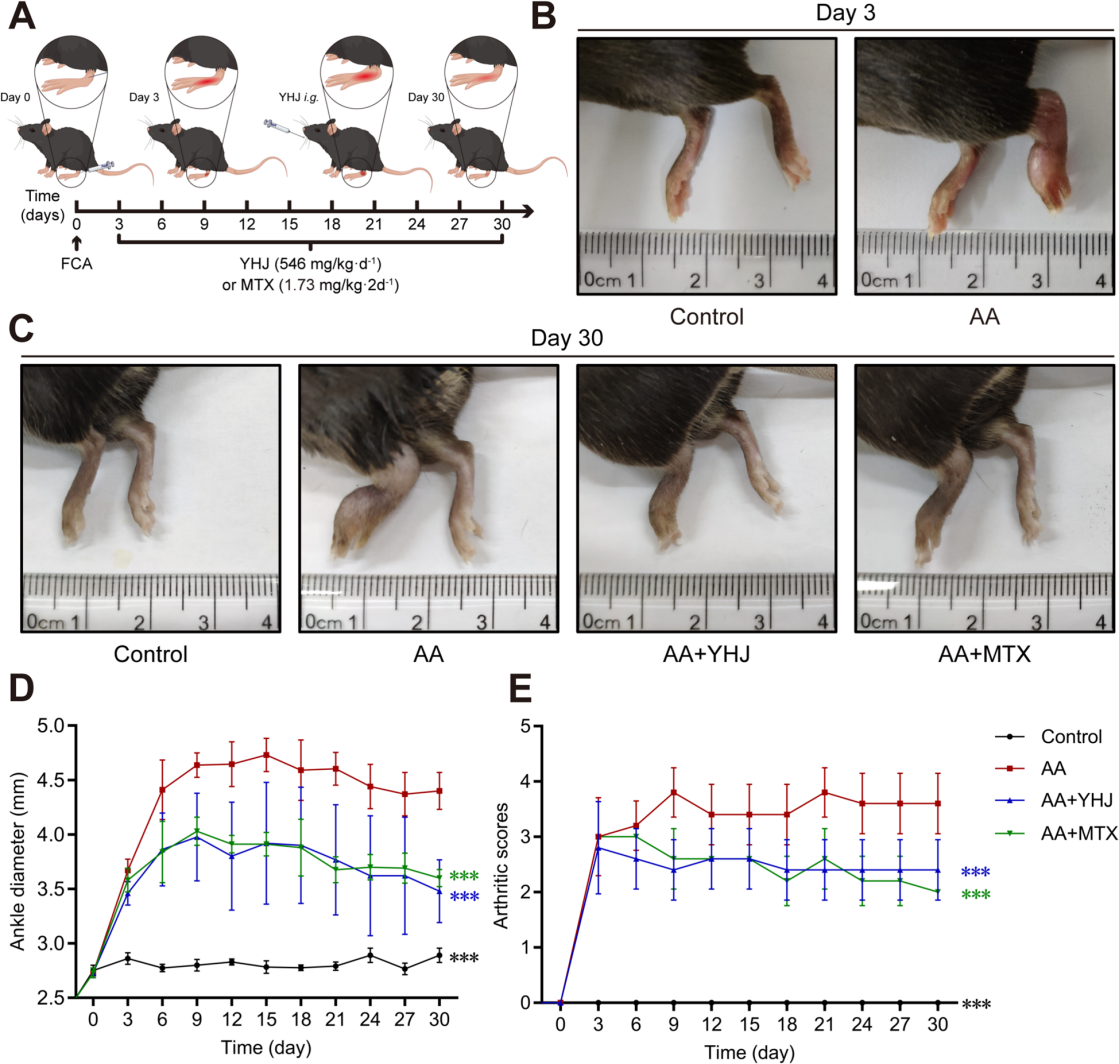
YHJ reduced ankle joint swelling and arthritis scores in AA mice. **A** Schematic diagram of the animal experiment. **B** Representative images of mouse hind paws on Day 3 after the FCA injection. **C** Representative images of the hind paws of the mice on Day 30. **D**, **E** Therapeutic effects of YHJ (546 mg·kg⁻^1^·d⁻^1^) and MTX in the initial experiment: **D** time course of ankle diameter, *n* = 5; **E** arthritis scores, *n* = 5. Data are presented as mean ± SD. In all panels: *AA* adjuvant arthritis model, *YHJ* Ya Han Jie, *MTX* methotrexate. Statistical significance: *p < 0.05, **p < 0.01, ***p < 0.001 versus the AA group

An AA mouse model was established to verify the anti-RA effect of YHJ in vivo **(**Fig. 1A**)**. In general, mice were considered arthritis models when the paw swelling and arthritis scores exceeded the thresholds. Three days after the injection of FCA into the mice, the joints of the mice showed typical redness and joint swelling locally. Thus, the mouse AA model was successfully constructed **(**Fig. 1B**)**. Daily oral YHJ and MTX treatments alleviated the degree of paw swelling and reduced arthritis scores in AA mice compared with the untreated AA group **(**Fig. 1C–E**)**. In addition, paw swelling was clearly more severe in the model mice than in the normal mice. Paw swelling was effectively relieved in AA mice after the oral administration of YHJ and MTX **(**Fig. 1C**)**. The above results indicated that YHJ significantly improved RA symptoms in AA mice. Additionally, YHJ inhibited arthritis in a dose-dependent manner (Supplementary Figure S1A) according to the ankle diameter (Supplementary Figure S1B) and arthritis scores (Supplementary Figure S1C). Further analysis was conducted with the high-dose treatment (YHJ).

### YHJ protects the bones and cartilage of AA mice and reduces inflammation levels

**Fig. 2:**
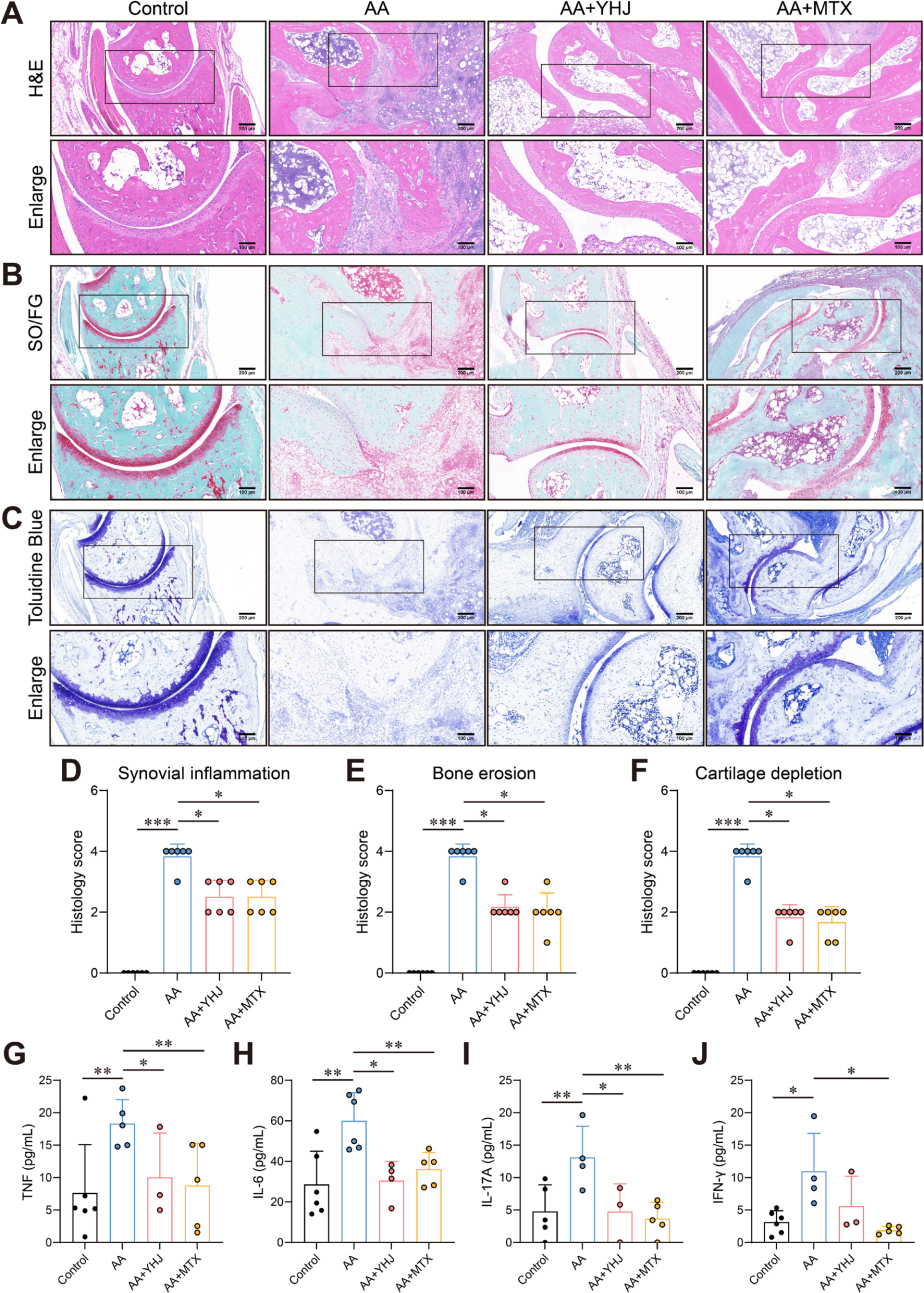
YHJ protected bone tissues in AA mice. **A**–**C** Representative images of hematoxylin and eosin (H&E), Safranin O-fast green and Toluidine blue staining of mouse joints. **D**–**F** Histology score of synovial inflammation, bone erosion, and cartilage depletion in mouse joints, *n* = 6. **G** The concentration of TNF in mouse serum,* n* ≥ 3. **H** The concentration of IL-6 in mouse serum,* n* ≥ 4. **I** The concentration of IL-17A in mouse serum,* n* ≥ 3. **J** The concentration of IFN-γ in mouse serum, *n* ≥ 3. The data are shown as the mean ± SD. *p < 0.05, **p < 0.01, and ***p < 0.001

H&E staining was performed to assess histopathological changes. In the AA group, the mice exhibited thickening of the joint cavity with significant inflammatory cell infiltration and apparent destruction of bone tissue and cartilage, indicating the successful induction of RA. Compared with the AA group, the YHJ and MTX treatment groups presented less inflammatory cell infiltration, a more complete joint structure, and less bone destruction **(**Fig. 2A, D, E**)**. Safranin O-fast green staining and Toluidine blue staining were subsequently performed to assess cartilage damage. Safranine O can stain glycosaminoglycans (GAGs) in cartilage red in this assay. Cartilage tissue contains many polysaccharides, such as chondroitin sulfate and hyaluronic acid. Toluidine blue is a commonly used cartilage stain in which its aromatic ring structure interacts with the hydroxyl or carboxyl groups in the polysaccharides in cartilage tissue to form a visibly stained complex. We found that the mice in the AA group had severe cartilage damage, with significantly attenuated or absent Safranin O and Toluidine blue staining, indicating a massive loss of GAGs and chondroitin sulfate and reflecting severe cartilage degradation **(**Fig. 2B, C, F**)**. After YHJ treatment, Safranin O and Toluidine blue staining was somewhat restored, suggesting that YHJ contributed to mitigating damage.

Inflammatory cytokines are crucial for coordinating systemic inflammation and exacerbating cartilage damage and bone erosion in individuals with RA [48]. We tested the effects of YHJ on the inflammatory response in AA mice by examining the levels of inflammatory cytokines closely associated with RA. The serum levels of TNF-α, IL-6, IL-17A, and IFN-γ were increased in AA mice compared with the control mice (Fig. 2G–J). However, treatment with YHJ and MTX reduced the levels of TNF-α, IL-6, IL-17A, and IFN-γ (Fig. 2G–J). These findings indicated that inflammatory cells were activated in AA mice, increasing the levels of inflammatory cytokines in the serum [49], and that YHJ inhibited this activation. These results indicated that YHJ exerted anti-inflammatory effects on AA mice.

To assess the safety of YHJ treatment, we performed histopathological analysis of liver and kidney tissues using H&E, PAS, and TUNEL staining. As shown in Supplementary Figure S2, no significant pathological changes—including necrosis, inflammatory infiltration, steatosis in the liver, or glomerular/tubular damage in the kidney—were observed in any of the YHJ-treated or MTX-treated groups compared to the Control group. Furthermore, PAS staining revealed no abnormalities in glycogen deposition or basement membrane integrity, and TUNEL staining showed no increase in apoptotic cells in either organ. These findings indicate that YHJ of the high dosage does not induce obvious hepatotoxicity or nephrotoxicity.

### Chemical profile of YHJ

**Fig. 3:**
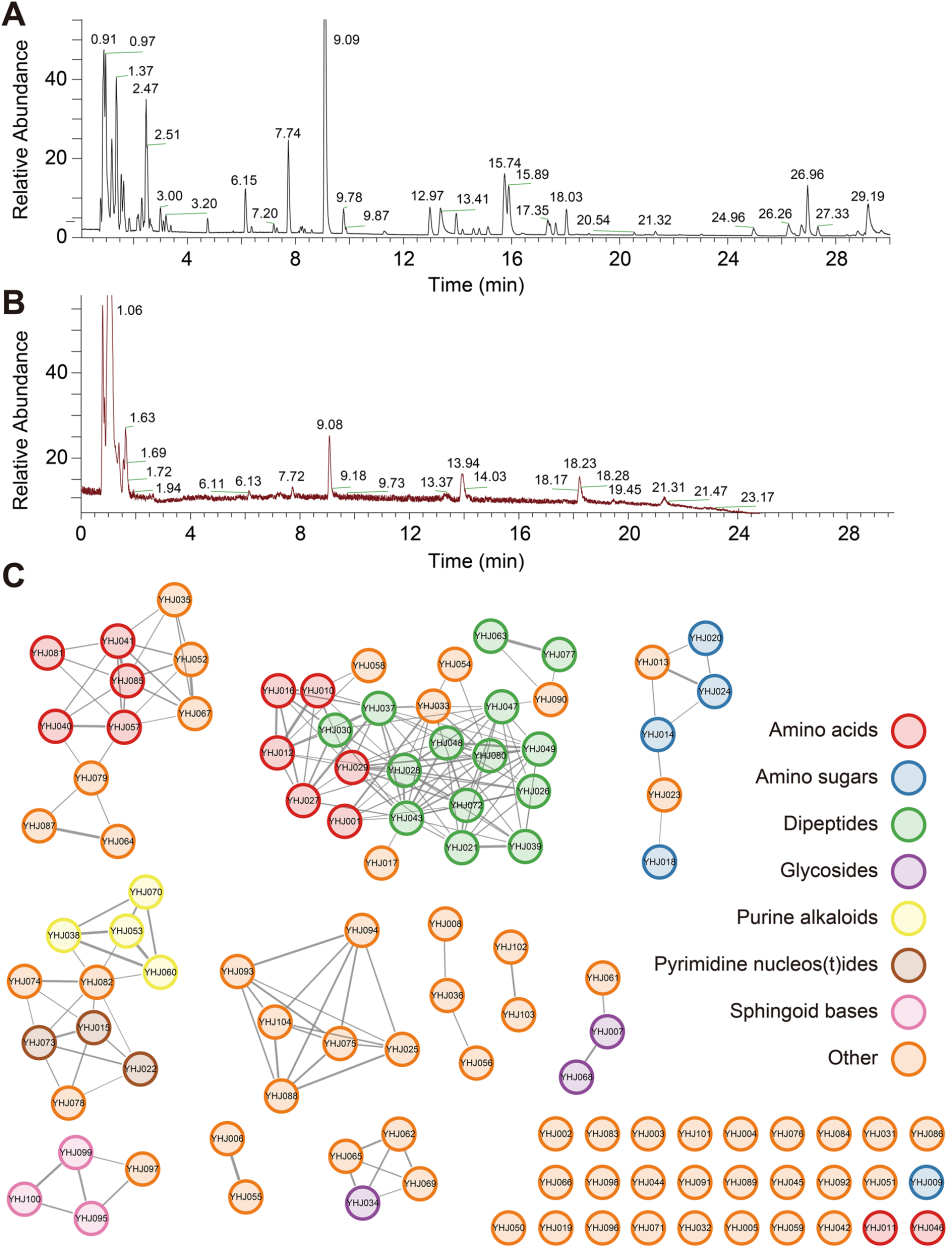
Total ion chromatograms of YHJ. **A** Positive ion mode. **B** Negative ion mode. **C** YHJ molecular network

In this study, the UPLC-Q-TOF MS/MS method was implemented to profile the chemical constituents of YHJ. Through a comprehensive identification process, a total of 104 natural small-molecule compounds were discerned. The corresponding total ion chromatograms (TICs) are shown in Fig. 3A, B, and detailed identification data are presented in Supplementary Table S2. Simultaneously, these compounds were chemically clustered using the GNPS platform to generate a mass spectrometry molecular network (Fig. 3C). According to the network analysis, YHJ comprises mainly amino sugars, glycosides, alkaloids, and a small amount of terpenoids. These components might be the basis of the anti-inflammatory activity of YHJ.

### Network pharmacology analysis

**Fig. 4:**
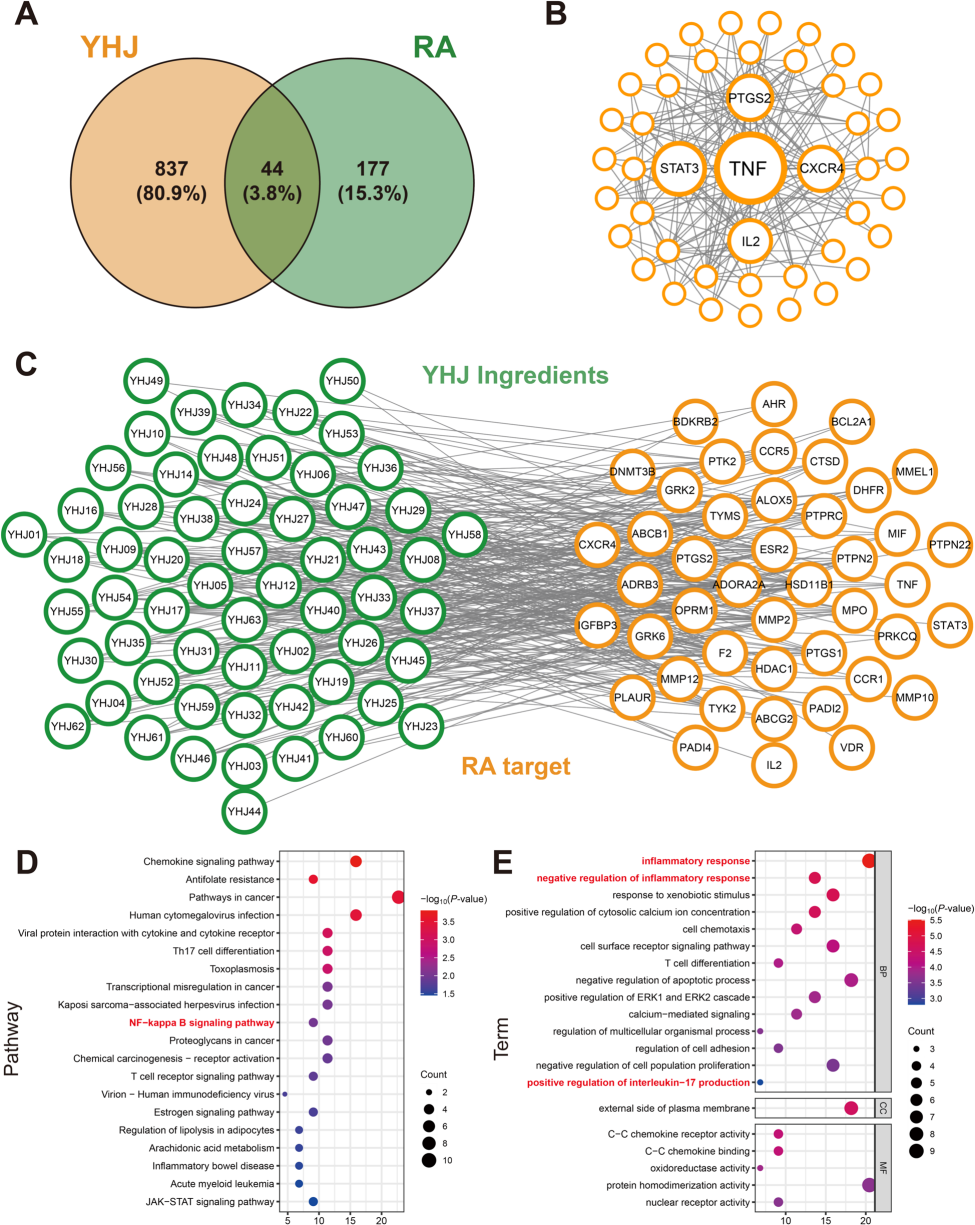
Network pharmacology analysis. **A** Venn diagram of YHJ-related targets and RA-related targets. **B** PPI network and clustering analysis of the intersecting targets of YHJ and RA. **C** YHJ chemical compound–target–RA network. **D** KEGG pathway enrichment analysis of the intersecting target genes of YHJ against RA; the top 20 pathways are listed. **E** Gene Ontology (GO) analysis of the intersecting target genes of YHJ against RA

We used the SwissADME tool to estimate the gastrointestinal absorption potential of YHJ components, and the components that the gastrointestinal tract could absorb were used as potential compounds for further research. Through the SwissTargetPrediction database, we obtained 881 targets of potential compounds. Moreover, 221 targets closely related to RA treatment were identified using the DisGeNET database. A total of 44 overlapping targets were subsequently identified by extracting common targets from both datasets (Fig. 4A). These shared targets were considered potential candidates for the treatment of RA. The PPI network was constructed using the STRING database to elucidate the main targets and potential mechanisms of action of YHJ in the treatment of RA (Fig. 4B). An in-depth analysis of the PPI network revealed five core targets, namely, TNF-α, STAT3, PTGS2, CXCR4, and IL2.

We further elucidated the relationships between the active components of YHJ and RA by constructing a network of YHJ compounds targeting RA with 110 nodes (Fig. 4C). The network consisted of 66 different compounds and 44 nonredundant targets interconnected by 332 edges, with an average composite node degree of 6.38. The four compounds with the highest degree values were YHJ05 (azidamfenicol), YHJ57 (DL tyrosine), YHJ12 (MFCD00059002) and YHJ63 (sucrose). Compounds with high degree values might play an essential regulatory role in treating RA. The intricate network highlighted the complex relationships between compounds and targets, suggesting that different components of YHJ exerted synergistic effects on the treatment of RA. YHJ achieved its therapeutic effect through multiple targets and pathways.

The KEGG enrichment analysis of common targets revealed that YHJ might regulate the chemokine signaling pathway, NF-κB signaling pathway, T-cell receptor signaling pathway, JAK-STAT signaling pathway and other pathways (Fig. 4D). The GO enrichment analysis revealed that YHJ might act on the external side of the plasma membrane to perform functions such as C–C chemokine receptor activity, C–C chemokine binding and oxidoreductase activity, thereby exerting biological effects such as an inflammatory response, negative regulation of the inflammatory response, response to xenobiotic stimulus and positive regulation of the cytosolic calcium ion concentration (Fig. 4E). These results suggested that YHJ exerted therapeutic effects on RA through these pathways.

### Characterization of YHJ-treated RA model mice via RNA-Seq analyses

**Fig. 5:**
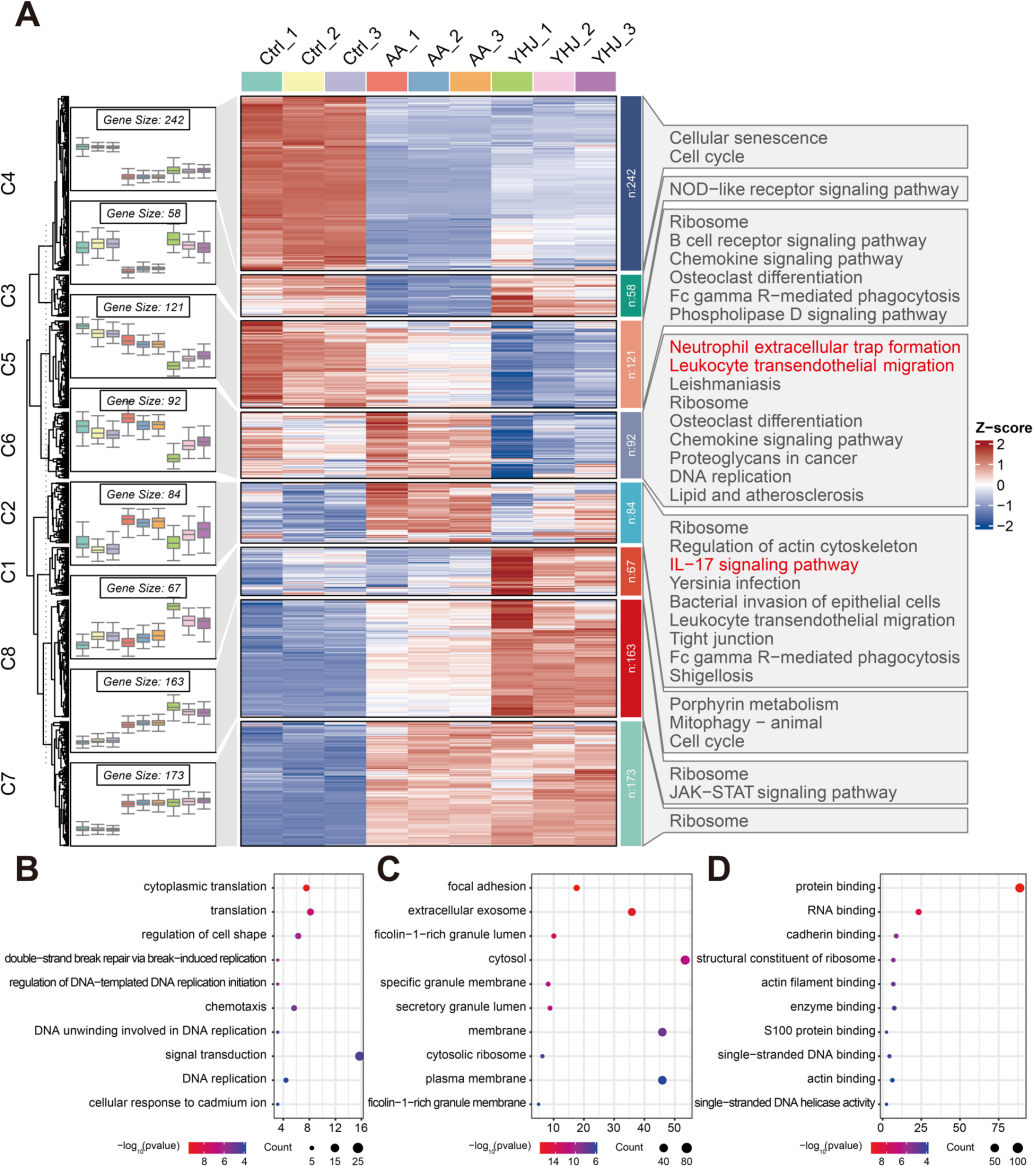
Clustering and enrichment analyses of the YHJ treatment for RA. **A** Cluster and visualization of gene expression matrix among Control, AA, and YHJ groups. Heatmap reveals eight clusters of genes, with each cluster exhibiting enrichment in KEGG analyses. Right-hand histogram shows the number of genes enriched in their respective terms. Heatmap and hierarchical clustering analysis of all genes. The color represents the normalized relative gene expression level. Red and blue represent upregulation and downregulation, respectively. **B** GO: Biological processes. **C** GO: Cellular components. **D** GO: Molecular functions

Transcriptome sequencing was performed on three RA samples from the Ctrl, AA, and YHJ groups using the Illumina high-throughput sequencing platform to further elucidate the mechanism of action of YHJ. The average number of raw reads obtained was 4.47 × 10^7^, and the average number of clean reads obtained after filtering was 4.34 × 10^7^. Compared with the control group, the AA group had 952 differentially expressed genes, of which 408 genes were significantly upregulated and 544 genes were significantly downregulated. Compared with the AA group, the YHJ group had 123 differentially expressed genes, 86 of which were significantly upregulated and 37 of which were significantly downregulated. Figure 5A shows the heatmap obtained after the clustering analysis of all genes, and a KEGG enrichment analysis was performed for different classes of genes. The results showed that YHJ significantly downregulated the expression of genes related to NET formation and leukocyte transendothelial migration. Moreover, compared with that in the AA group, the expression of genes related to the IL-17 signaling pathway was downregulated. The GO functional enrichment analysis revealed that, compared with those in the AA group, the differentially expressed genes in the YHJ group were enriched mainly in molecular functions such as protein binding, RNA binding, and cadherin binding (Fig. 5B–D). YHJ regulated biological processes such as translation, cytoplasmic translation, and the regulation of cell shape. In terms of cell composition, the differentially expressed genes were mainly enriched in focal adhesion, the extracellular exosome, and the ficolin-1-rich granule lumen. We hypothesized that YHJ could act on the chemokine signaling pathway and NF-κB signaling pathway, affect the expression of genes related to NET formation and leukocyte transendothelial migration, and play a role in the treatment of RA.

### YHJ inhibits neutrophil infiltration and NET formation in joints

**Fig. 6:**
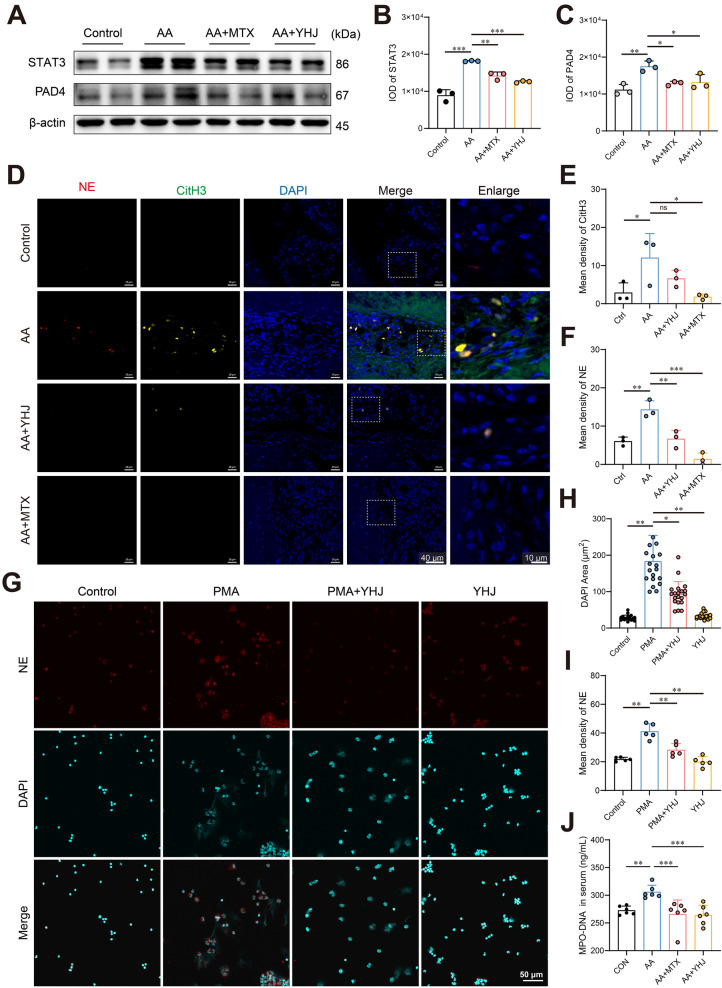
YHJ inhibits neutrophil migration and NET formation. **A** Western blot analysis of protein expression in joint tissues. **B**–**C** The quantification of expression level of STAT3 and PAD4 in joint tissues, *n* = 3. **D** Representative images of triple-labeled NE (red) and CitH3 (green) immunofluorescence showing the colocalization of NE and CitH3 (white arrows). **E** The mean density of CitH3 in (A), *n* = 3. **F** The mean density of the NE in (A), *n* = 3. **G** IF staining was performed to determine the level of NE in neutrophils. Representative images are shown. **H** The nuclear areas in **C**, *n* ≥ 3. **I** The mean density of the NE in **D**, *n* = 5. **J** The serum levels of MPO-DNA complexes, *n* = 6. The data are shown as the mean ± SD. *p < 0.05, **p < 0.01, and ***p < 0.001

Neutrophils are abundant in the inflamed joints of RA patients, especially in the early stages of the disease, and can release NETs locally [19]. In patients with RA, both neutrophil elastase (NE) and PAD4 participate in the formation of NETs. PAD4 can catalyze the citrullination of histones, such as citrullinated histone H3 (CitH3). These citrullinated histones and NE are enriched in NETs, which can promote the autoimmune response and inflammatory process [50, 51]. Therefore, we examined whether YHJ regulates PAD4 expression. Western blot analysis showed that YHJ significantly downregulated PAD4 protein levels in joint tissues (Fig. 6A, C). A previous study reported that treatment with the PAD4 inhibitor GSK484 alleviated disease severity in the mice with collagen-induced arthritis [52]. Consistently, in our AA mouse model, GSK484 treatment significantly ameliorated joint swelling and reduced arthritis scores (Supplementary Figure S3). Furthermore, network pharmacology analysis identified STAT3 as a core target of YHJ in RA treatment (Fig. 4B). To validate this prediction, we examined STAT3 expression and found that YHJ also significantly downregulated STAT3 protein levels in joint tissues (Fig. 6A, B).

Therefore, we first examined the effect of YHJ on NET formation in vivo by performing multiplex immunohistochemistry (mIHC) experiments. Consistent with the transcriptome data, NE, PAD4 and CitH3 were highly expressed in joint tissues of the AA group, and their expression was significantly reduced by YHJ treatment **(**Fig. 6D–F**)**. The results showed that YHJ significantly inhibited the infiltration of neutrophils into the joint area and the local formation of NETs.

We further explored the effect of YHJ on NETs in vitro. We isolated and purified primary neutrophils from the mice and then added phorbol 12-myristate 13-acetate (PMA) to induce NETosis in vitro. After PMA stimulation, the cells exhibited an expanded morphology accompanied by a significant increase in the NE signal, indicating that the neutrophils were activated and that NE was released. In the PMA-YHJ cotreatment group, the neutrophil morphology was still slightly activated, but NE signaling was reduced compared with that in the PMA-treated group, indicating that YHJ inhibited PMA-induced neutrophil activation and NE release (Fig. 6G–I).

To provide a systemic and quantitative measure of NETosis, we assessed serum levels of MPO-DNA complexes, a specific circulating marker of NETs. Consistent with the histological findings, AA mice exhibited a significant elevation in MPO-DNA levels, which was markedly reduced by YHJ treatment (Fig. 6J). This confirms that YHJ potently inhibits NETosis not only locally in the joints but also at the systemic level.

### YHJ inhibits the activation of the TNF-α/NF-κB signaling pathway in joints

**Fig. 7:**
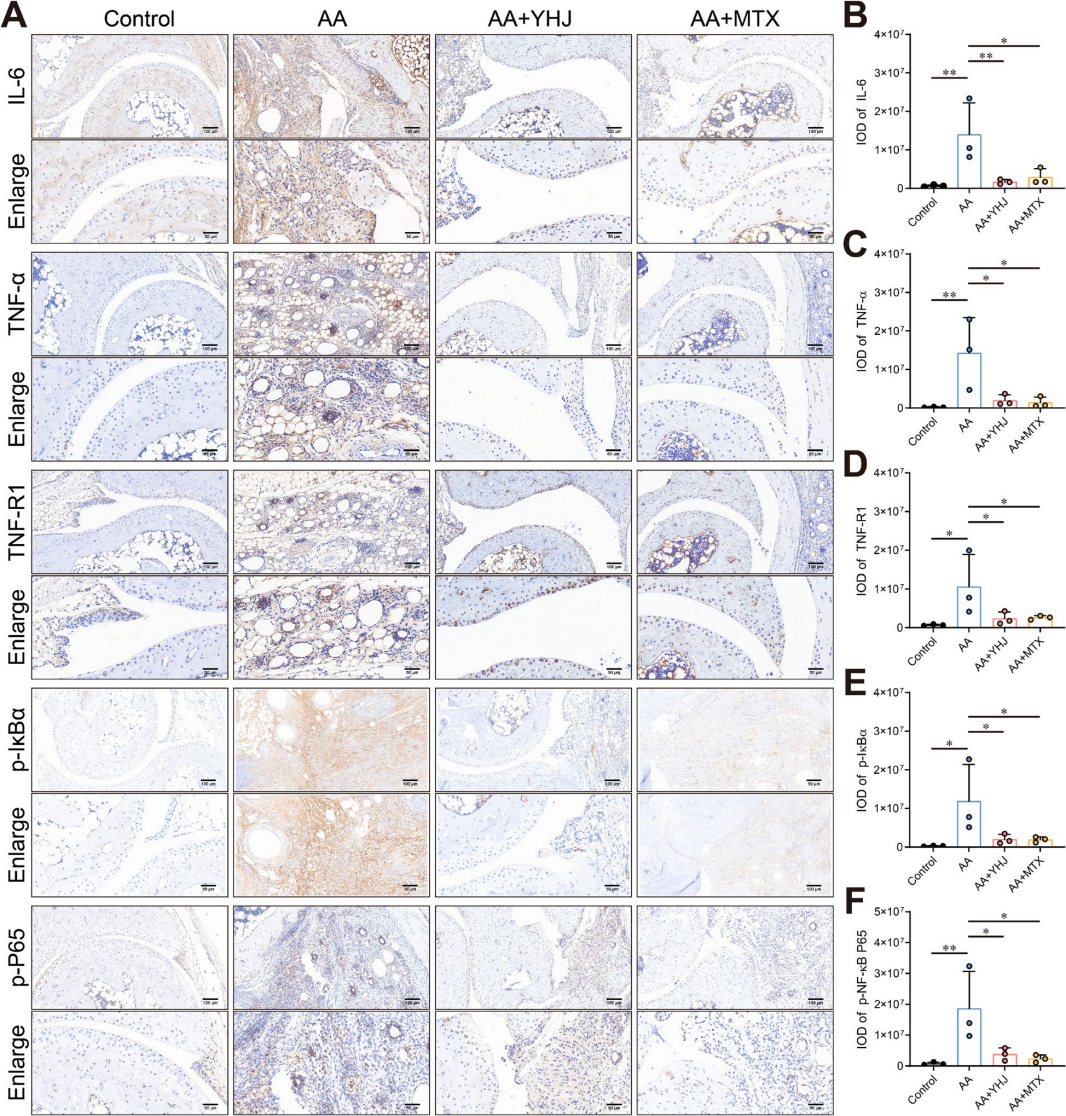
YHJ regulated the TNF-α/NF-κB signaling pathway in AA mice. **A** Representative images of immunohistochemical staining for TNF-α, TNF-R1, IL-6, p-IκBα and p-P65. **B** Quantification of the IL-6 levels in (A) using ImageJ software, *n* = 3. **C** Quantification of the TNF-α levels in **A** using ImageJ software, *n* = 3. **D** Quantification of the TNF-R1 levels in **A** using ImageJ software, *n* = 3. **E** Quantification of the p-IκBα levels in **A** using ImageJ software, *n* = 3. **F** Quantification of the p-P65 levels in **A** using ImageJ software, *n* = 3. The data are shown as the mean ± SD.*p < 0.05, **p < 0.01, and ***p < 0.001

According to the above network and molecular docking analyses, the main targets of YHJ in treating RA were significantly enriched in the TNF-α/NF-κB signaling pathway, which plays a crucial role in immune-mediated inflammatory diseases. We next experimentally examined the effect of YHJ on the TNF-α/NF-κB pathway to verify its anti-RA mechanism. In particular, we performed IHC staining to detect the levels of IL-6, TNF-α, TNF-R1, p-IκBα and p-P65 (p-NF-κB) in the synovial tissue of the control and AA mice. As shown in Fig. 7, the levels of IL-6, TNF-α, TNF-R1, p-IκBα and p-P65 in the synovium of AA mice were significantly higher than those in the control mice. In contrast, YHJ treatment significantly reduced the levels of these proteins in arthritic mice.

### Functional validation of the TNF-α/NF-κB axis as a driver of NETosis

The aforementioned data suggest that YHJ alleviates RA by suppressing the TNF-α/NF-κB axis and subsequent NETosis. To validate that this axis is functionally capable of driving NETosis, we detected the changes of neutrophil induced by TNF-α in vitro.

**Fig. 8:**
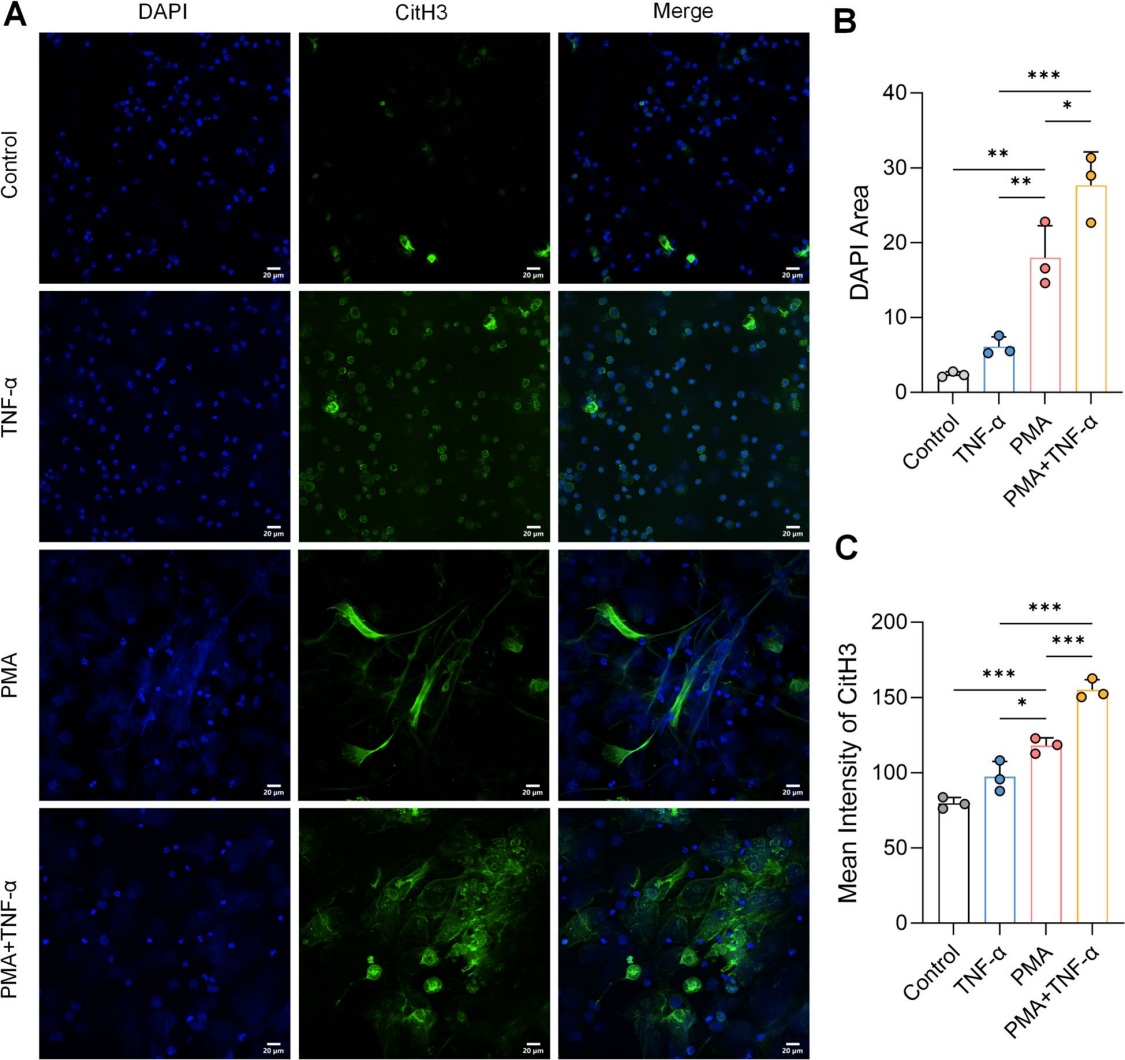
Exogenous activation of the TNF-α/NF-κB axis potentiates NETosis in vitro. **A** Representative immunofluorescence images showing citrullinated histone H3 (CitH3, green) and nuclei (DAPI, blue) in primary mouse neutrophils under the indicated treatments: Control (medium only), TNF-α (50 ng/mL), PMA (100 nM), and PMA + TNF-α (100 nM + 50 ng/mL). **B** Quantitative analysis of the mean fluorescence intensity of CitH3 per field, *n* = 3. **C** Quantitative analysis of the DAPI area per field, representing nuclear expansion, *n* = 3. Data are presented as mean ± SD. *p < 0.05, **p < 0.01, and ***p < 0.001

Morphological analysis of primary mouse peritoneal neutrophils revealed distinct stages of NETosis under different treatments (Fig. 8A). In the Control group, DAPI staining showed small, dense, and well-defined nuclei (typically 2–5 lobes), and CitH3 signal was barely detectable, reflecting a baseline state. Stimulation with TNF-α alone induced early or mild NETotic changes: nuclei began to swell, accompanied by the emergence of punctate CitH3 signals. In contrast, stimulation with PMA alone triggered robust NETosis, characterized by nuclear membrane rupture, chromatin decondensation, and strong, diffuse CitH3 signals forming web-like structures. Crucially, co-stimulation with recombinant TNF-α protein—an exogenous activator of the NF-κB pathway—further potentiated PMA-induced NETosis. This was evidenced by more extensive chromatin spreading and a significant increase in CitH3 fluorescence intensity compared to the PMA-only group (Fig. 8C). In parallel, the DAPI area, reflecting nuclear expansion and chromatin decondensation, was also significantly larger in the PMA + TNF-α group than in the PMA group (Fig. 8B).

These results demonstrate that enhanced signaling through the TNF-α/NF-κB axis can directly exacerbate the NETotic response at both morphological and quantitative levels. Therefore, the therapeutic inhibition of this axis by YHJ, as demonstrated in vivo, contributes to its anti-NETotic and anti-arthritic effects.

### Molecular dynamic simulation

**Fig. 9:**
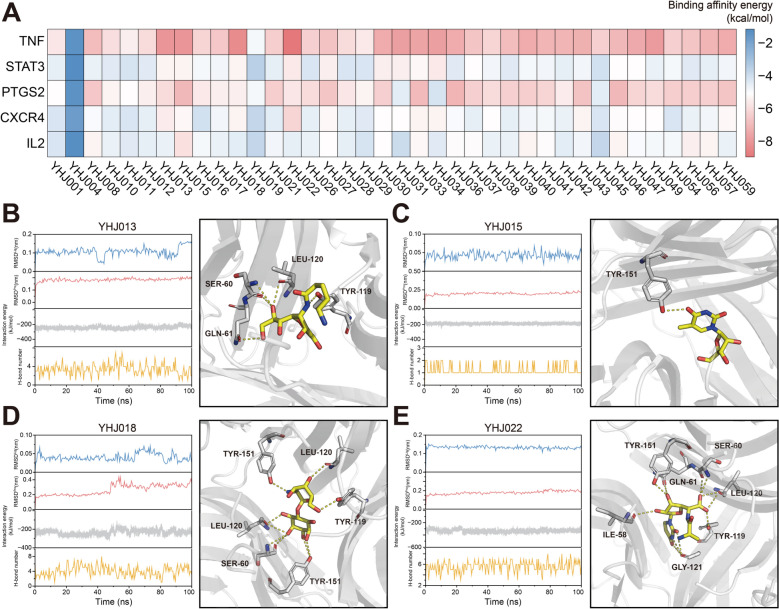
Molecular docking and molecular dynamic simulation. **A** Heatmap of the binding affinities between the potential compounds in YHJ and core targets of RA. Binding patterns between YHJ013 (**B**), YHJ015 (**C**), YHJ018 (**D**), YHJ022 (**E**) and TNF-α

We performed molecular docking of the potential compounds in YHJ with core targets in the PPI network. The results revealed that the potential compounds had negative affinity values for these targets, indicating that intermolecular interactions could occur spontaneously (Fig. 9A). All of them had a low binding affinity for TNF-α. The four compounds with the lowest energy were YHJ013 (−7.9 kcal/mol), YHJ015 (−7.9 kcal/mol), YHJ018 (−8.2 kcal/mol) and YHJ022 (−8.9 kcal/mol).

TNF-α plays an important role in RA. In individuals with RA, TNF-α activates synovial fibroblasts, leading to the excess production of proteases and matrix metalloproteinases (MMPs) that degrade collagen and proteoglycans, leading to cartilage and bone destruction and joint erosion [53]. In addition, TNF-α promotes the pathological progression of RA by activating osteoclasts and inducing synovial hyperplasia and angiogenesis [54]. Therefore, the inhibition of TNF-α can attenuate the pathological progression of RA.

We further investigated the interactions of these four compounds with TNF-α by using molecular dynamics simulations. The RMSD was used as an index to evaluate the stability of the ligand and receptor throughout the simulation. All four compounds reached a steady state within a simulation time of 100 ns, and the RMSDs of the proteins and small molecules were within a small range. Thus, the conformational change in the protein was small at the prescribed time scale. In addition, all the compounds could form stable hydrogen bonds with TNF-α, with an average binding energy of less than −200 kJ/mol **(**Fig. 9B–E**)**. The above results indicated that these compounds were able to interact stably with TNF-α, which might be the basis for the pharmacological effects of YHJ.

## Discussion

RA is a chronic autoimmune disease characterized by excessive immune system activation, leading to the recruitment of inflammatory cells such as neutrophils, T cells, macrophages, and fibroblasts into the joint space. This process results in joint swelling, redness, and, eventually, damage to the cartilage and bone. In the early stages of RA, neutrophils play a pivotal role by becoming activated after migrating into the joint cavity, suggesting their key involvement in the initial inflammatory events of RA [18, 55]. Dai medicine, with its unique approach to understanding, diagnosing, and treating diseases, has produced promising clinical outcomes. It offers a comprehensive system for managing RA, improving treatment efficacy while minimizing side effects. YHJ, a commonly used prescription in Dai medicine, has significant therapeutic effects on osteoarthritis and joint-related diseases.

In this study, we used the mouse FCA-induced AA model and a multiomics approach to investigate the mechanisms through which YHJ treats RA. The results showed that YHJ significantly alleviated RA symptoms in the joints of AA mice, including inflammatory cell infiltration, cartilage erosions and bone destruction. Notably, these therapeutic effects were dose-dependent. Additionally, YHJ effectively reduced the levels of key inflammatory factors, such as TNF, IL-6, IL-17A, and IFN-γ, in the serum, thereby alleviating inflammation. Preliminary safety assessment further revealed no signs of hepatotoxicity at the therapeutic doses, highlighting its potential for safe clinical application.

To further analyze the potential mechanism, UPLC-Q-TOF MS/MS technology, and the network pharmacology analysis were performed. We identified 104 bioactive components in YHJ, including amino sugars, glycosides, alkaloids, and some terpenoids. The network pharmacology analysis revealed that TNF-α is the core target of YHJ in RA treatment. TNF-α is a crucial cytokine in the inflammatory cascade of RA and is produced by chondrocytes, monocytes, osteoblasts, and synovial tissue. It plays a key role in amplifying and perpetuating the RA disease process by inducing the production of proinflammatory cytokines and chemokines [56, 57]. Further KEGG and GO enrichment analyses indicated that YHJ affects key signaling pathways involved in RA, particularly the chemokine and NF-κB pathways. Chemokine signaling contributes to the expression of chemokines such as CCL2, CCL4, c-Jun, and c-Fos in peripheral blood leukocytes and synovial tissue, facilitating the recruitment of leukocytes to RA-affected joints [58]. The NF-κB pathway, another critical pathway involved in RA, is activated by stimuli such as TNF-α, IFN-γ, and LPS, leading to IκB degradation and subsequent activation of NF-κB. The translocation of NF-κB into the nucleus regulates the transcription of inflammatory genes, including TNF-α, IL-1β, IL-6, and inducible nitric oxide synthase (iNOS) [59, 60].

The RNA sequencing analysis was also performed to provide a comprehensive view of the differentially expressed genes induced by YHJ, revealing the significant downregulation of genes related to neutrophil extracellular trap (NET) formation and leukocyte transendothelial migration. NETs consistently activate the surrounding cells to release inflammatory cytokines such as IL-1β, IL-6, and TNF-α, which can exacerbate joint tissue damage and RA progression [61]. Malamud et al. identified MICL as an inhibitory receptor for NETs, whose dysfunction exacerbates arthritis [62]. We also revealed that inhibition of NETosis with GSK484 alleviated AA murine model, further validating NETs as a therapeutic target in RA. In vivo, YHJ treatment significantly downregulated the expression of NE, CitH3 and PAD4 in the joints, as well as MPO-DNA complex in the serum. The in vitro experiments also showed that YHJ could inhibit NET formation. Therefore, YHJ treatment is proved capable of suppressing NETs in RA.

A hallmark of RA is chronic synovial inflammation, which is characterized by the extensive infiltration of activated leukocytes, leading to cartilage and bone destruction [63]. IHC assays and WB results of STAT3 revealed that YHJ inhibited the activation of the TNF-α/NF-κB pathway More importantly, we demonstrated that exogenous activation of the TNF-α/NF-κB pathway with recombinant TNF-α protein could potentiate PMA-induced NETosis. This critical finding validates that the TNF-α/NF-κB axis is not merely associated with but is a functional driver of NETosis. IL-6 was also reported to drive PAD4 expression in neutrophils during early arthritis [64]. These findings suggest that YHJ alleviates RA by inhibiting neutrophil infiltration and NET formation and that its therapeutic effects are causally related to the suppression of the TNF-α/NF-κB signaling pathway.

## Conclusions

**Fig. 10:**
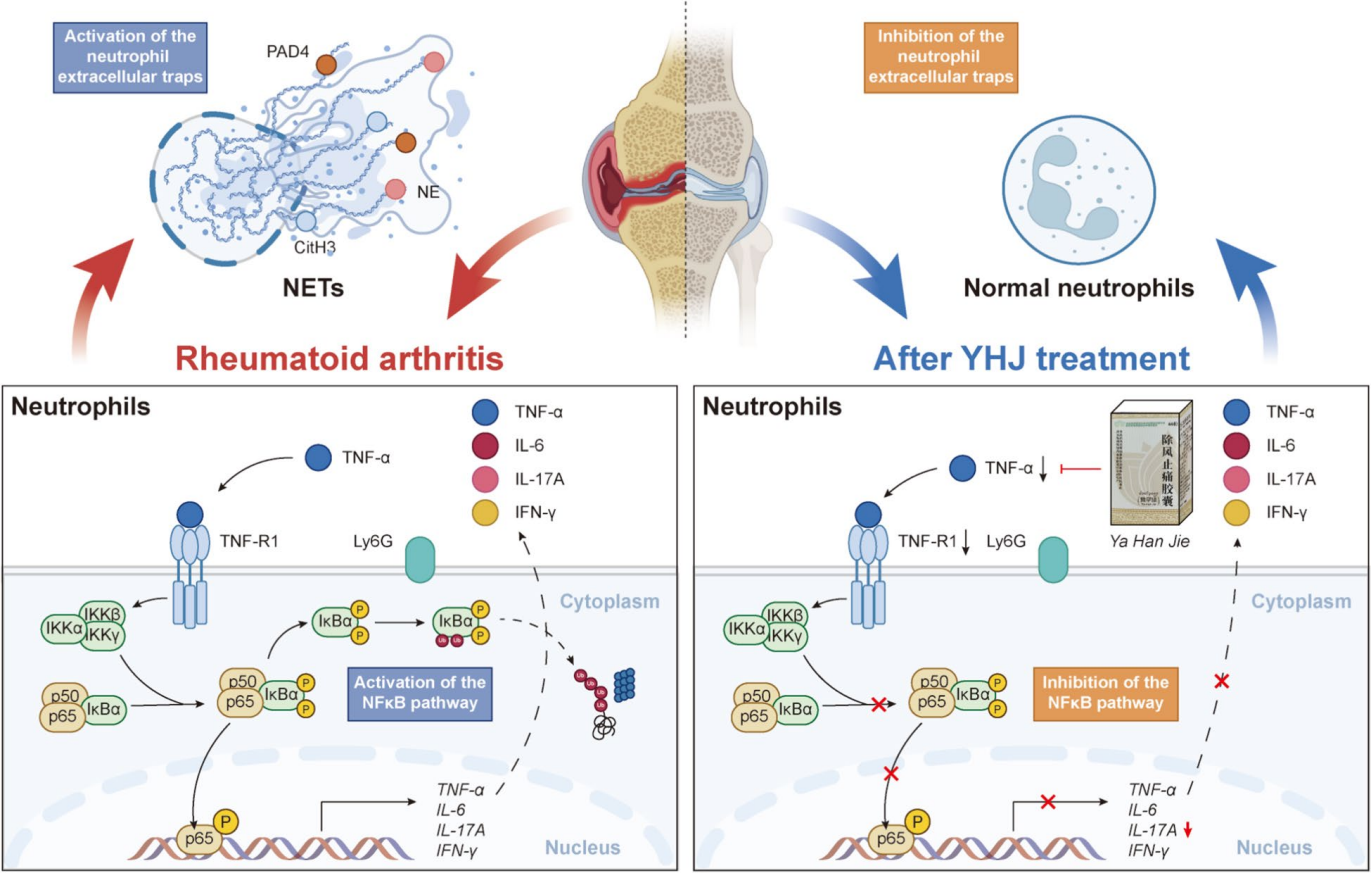
Schematic diagram of the mechanism of action. YHJ inhibits neutrophil extracellular trap formation by suppressing the TNF-α/NF-κB signaling pathway

YHJ exerts notable therapeutic effects on AA mice by inhibiting the activation of NF-κB, reducing the expression of proteins involved in NET formation, and suppressing both NET formation and the release of inflammatory factors both in vitro and in vivo (Fig. 10).

## Supplementary Information

Additional file 1

Additional file 2

Additional file 3

## Data Availability

The data of this manuscript are available from the corresponding author upon reasonable request. The transcriptome raw data in IGT research of this study are publicly available in the NCBI Sequence Read Archive under the accession code PRJNA1222308.

## Abbreviations

AA: Adjuvant arthritis
ACPA: Anti-citrullinated peptide antibodies
CBA: Cytometric Bead Array
CitH3: Citrullinated histone H3
H&E: Hematoxylin and eosin staining
IFN-γ: Interferon-γ
IHC: Immunohistochemical staining
IL-10: Interleukin-10
IL-17A: Interleukin-17A
IL-1β: Interleukin-1 beta
IL-2: Interleukin-2
IL-4: Interleukin-4
IL-6: Interleukin-6
MPO: Myeloperoxidase
MTX: Methotrexate
NE: Neutrophil elastase
NETs: Neutrophil extracellular traps
|NF-κB: Nuclear factor kappa-B
PAD4: Protein-arginine deiminase type-4
PDB: Protein Data Bank
PPI: Protein–protein interaction
RA: Rheumatoid Arthritis
SO/FG: Safranin O-Fast Green
TNF-α: Tumor necrosis factor-alpha
UFLC-Q-TOF MS/MS: Ultra-fast liquid chromatography-quadrupole time of flight tandem mass spectrometry
YHJ: Ya Han Jie
