# Supplementary material for: Ya Han Jie ameliorates adjuvant-induced arthritis by inhibiting the NF-κB/NETosis/inflammation axis
Source: Chin Med. 2026 May 22;21:140. doi: 10.1186/s13020-026-01392-2 (PMC13195958; doi:10.1186/s13020-026-01392-2)
Supplement: Supplementary file 1 — Additional file 1 [file 13020_2026_1392_MOESM1_ESM.docx]

###
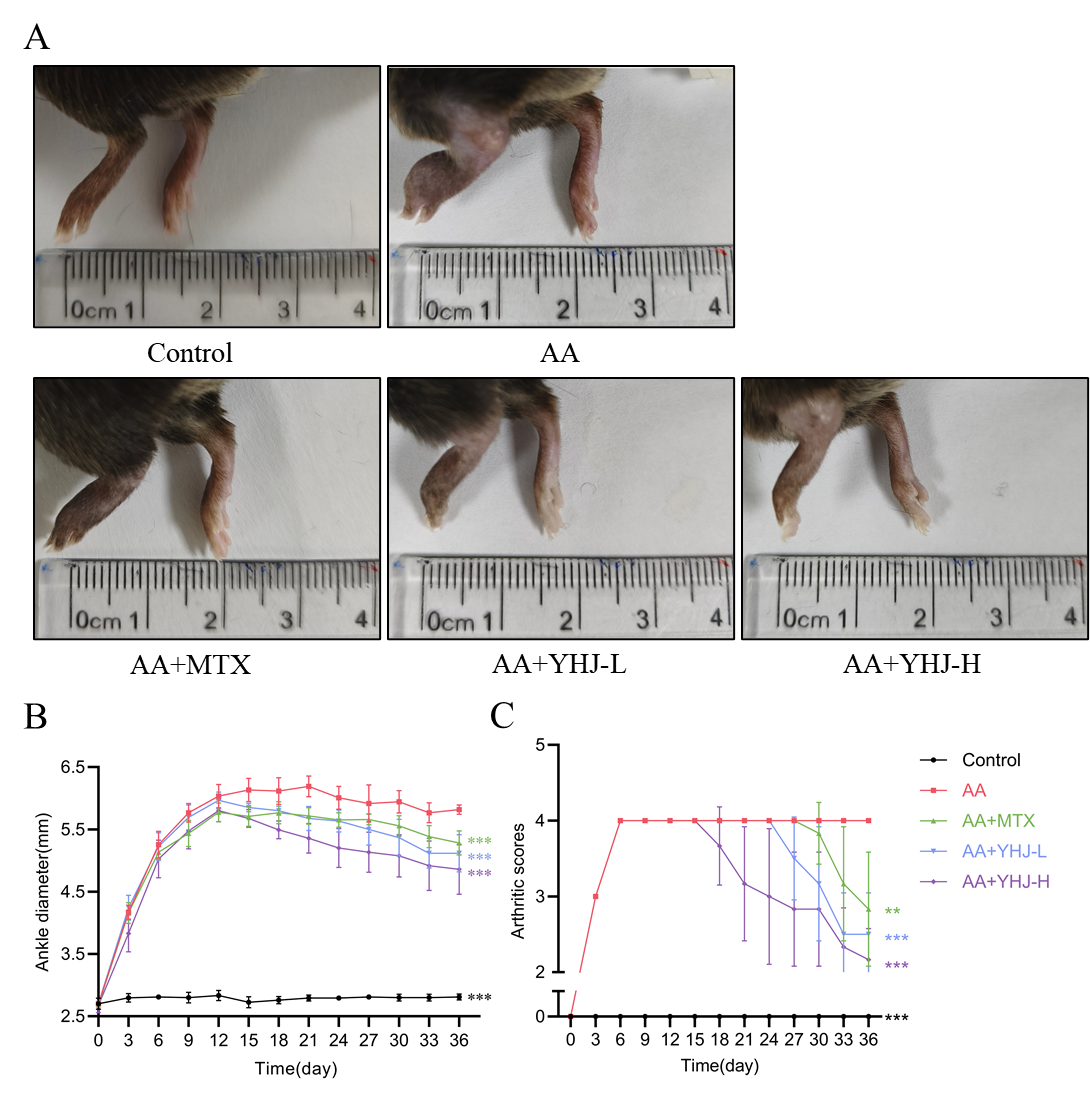
****Supplementary Figure****

**Supplementary Figure S1.** **YHJ reduced ankle joint swelling and arthritis scores in AA mice in a dose-dependent manner.** (A) Representative images of hind paws on the Day 36 of the experiment. (B, C) Dose-response assessment of YHJ from an independent, extended-duration experiment: (B) time course of ankle diameter over 36 days; (C) corresponding arthritis scores. YHJ-L, YHJ treatment with low-dose; YHJ-H, YHJ treatment with high-dose. Data are mean ± SD (*n =* 6 mice per group). **p* < 0.05, ***p* < 0.01, ****p* < 0.001 versus the AA group.

**
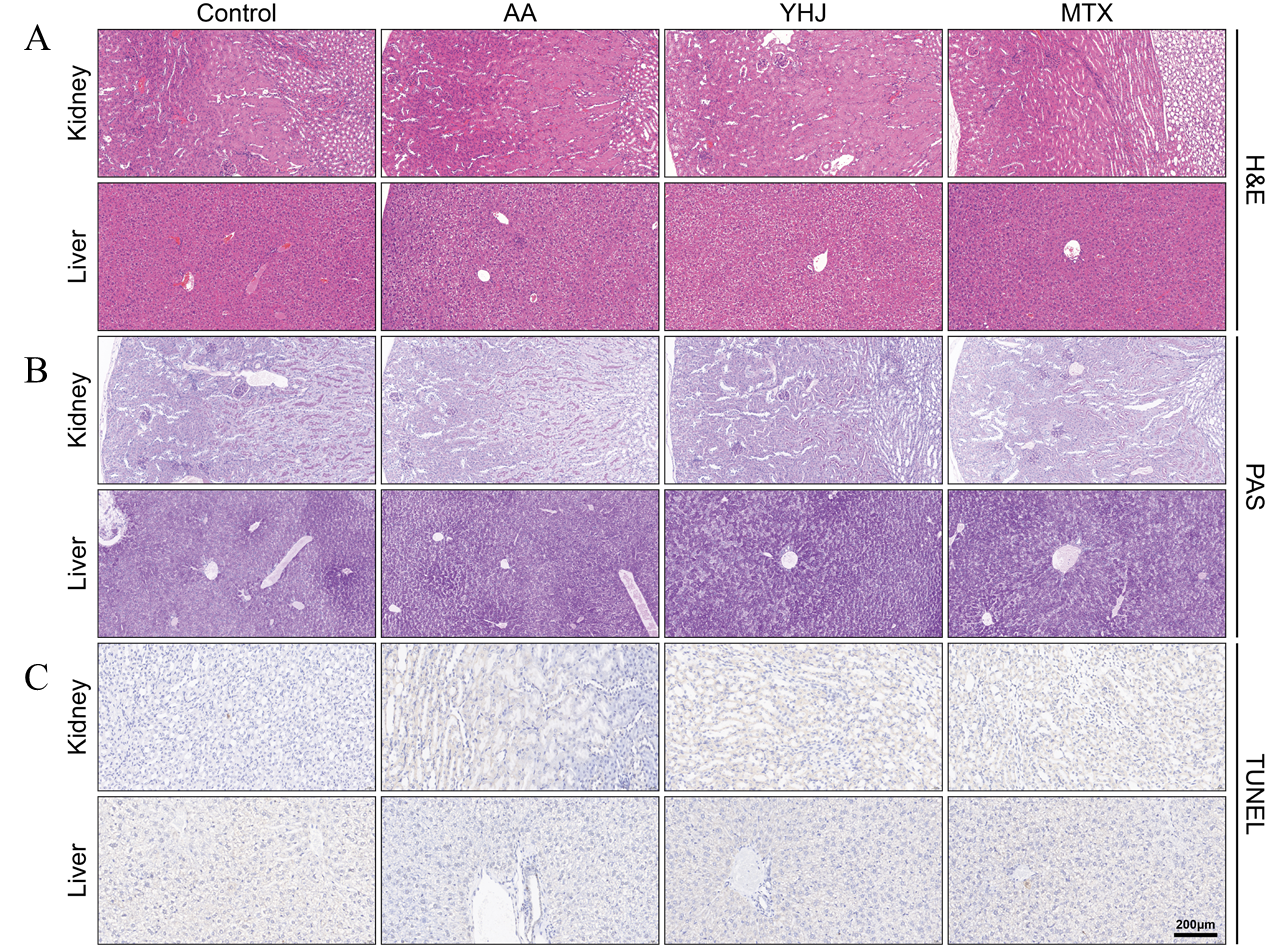
**

**Supplementary Figure S2. Safety assessment of YHJ in liver and kidney tissues of AA mice.** (A) H&E staining. (B) PAS staining. (C) TUNEL assays.

**
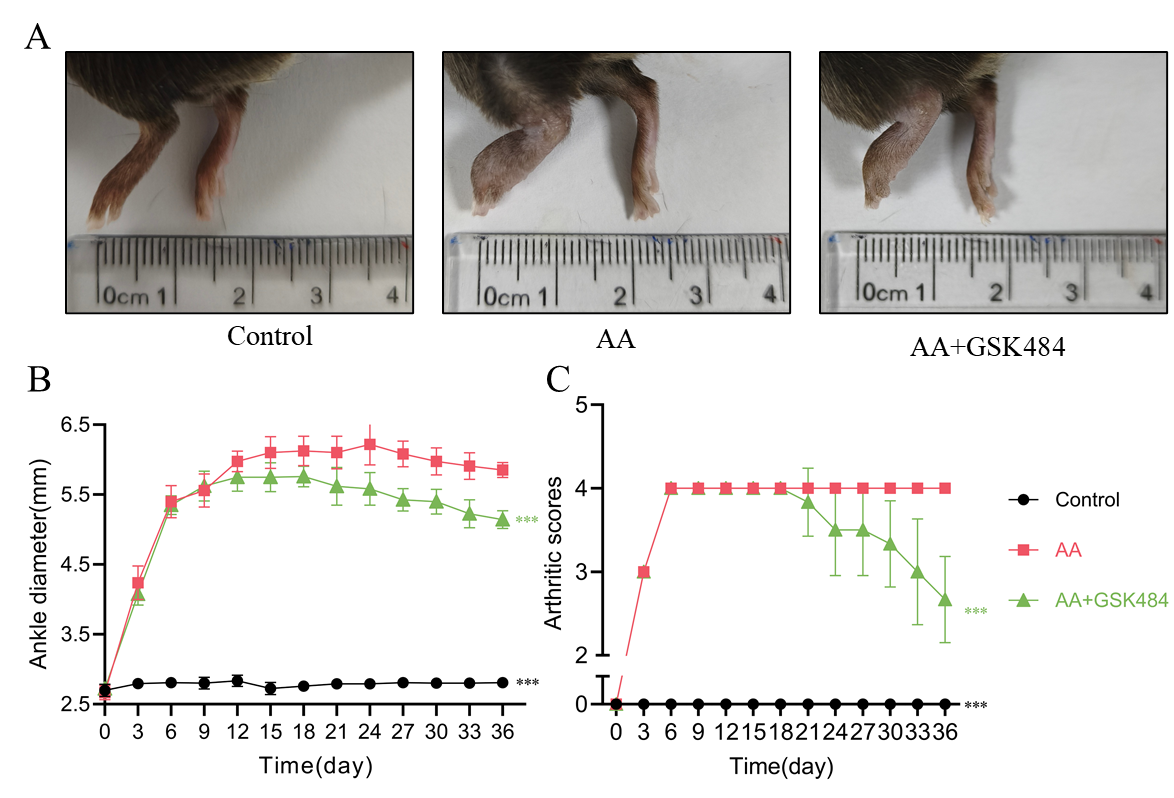
**

**Supplementary Figure S3. The therapeutic effects of PAD4 inhibitor GSK484 in AA mice.** (A) Representative images of hind paws on the final day (day 36) of the experiment. (B) Ankle joint diameter measurement over time. (C) Arthritis score assessment. Data are presented as mean ± SD; *n*=6 per group. **p* < 0.05, ***p* < 0.01, ****p* < 0.001 versus the AA group.
