# Supplementary material for: Ya Han Jie ameliorates adjuvant-induced arthritis by inhibiting the NF-κB/NETosis/inflammation axis
Source: Chin Med. 2026 May 22;21:140. doi: 10.1186/s13020-026-01392-2 (PMC13195958; doi:10.1186/s13020-026-01392-2)
Supplement: Supplementary file 2 — Additional file 2 [file 13020_2026_1392_MOESM2_ESM.docx]

**Supplementary Table S1. The criteria for the arthritis score**

| Score | Standard of the arthritis score |
| --- | --- |
| 0 | Normal paw |
| 1 | Inflammation and swelling of one toe |
| 2 | Inflammation/redness in more than one toe |
| 3 | Inflammation and swelling of the entire paw |
| 4 | Severe inflammation and swelling of the entire paw or ankylosed paw |
